# Tailoring Epoxy Resin Foams by Pre-Curing with Neat Amine Hardeners and Its Derived Carbamates

**DOI:** 10.3390/polym13081348

**Published:** 2021-04-20

**Authors:** Christian Bethke, Sebastian Manfred Goller, Uy Lan Du Ngoc, Simon Tino Kaysser, Volker Altstädt, Holger Ruckdäschel

**Affiliations:** 1 Department of Polymer Engineering, University of Bayreuth, Universitaetsstrasse 30, 95447 Bayreuth, Germany; christian.bethke@uni-bayreuth.de (C.B.); sebastian.goller@uni-bayreuth.de (S.M.G.); Du.Uy-Lan@uni-bayreuth.de (U.L.D.N.); kaysser@comprisetec.de (S.T.K.); altstaedt@uni-bayreuth.de (V.A.); 2 Bavarian Polymer Institute and Bayreuth Institute of Macromolecular Research, University of Bayreuth, Universitaetsstrasse 30, 95447 Bayreuth, Germany

**Keywords:** epoxy foam, thermoset foam, carbamate, pre-curing, batch foaming, rheology, DSC

## Abstract

The use of amine-based carbamates with their dual function, acting as amine curing agents and CO_2_ blowing agents after their decomposition without by-products, are promising for ecofriendly epoxy foams as high-performance materials. However, controlling cell morphology requires a proper adjustment of the viscosity at the foaming step. The viscosity is altered not only by blending neat amine and its derived carbamate at a fixed pre-curing time, but also by changing the pre-curing time at a fixed blend ratio. Within this study, diglycidylether of bisphenol A (DGEBA) epoxy resin is mixed with different blend ratios of isophorone diamine (IPDA) and its derived carbamate (B-IPDA). The systems are characterized by DSC and rheology experiments to identify the pre-curing effects on the derived epoxy foams. Epoxy foams at a blend ratio of 30/70_w_ IPDA/B-IPDA showed the best foam morphology and an optimum Tg compared to other blend ratios. Furthermore, it was found that both pre-curing times, 2 h and 3 h, for the 30/70_w_ IPDA/B-IPDA system reveal a more homogeneous cell structure. The study proves that the blending of neat amine and carbamate is beneficial for the foaming performance of carbamate systems.

## 1. Introduction

Foamed epoxy materials exhibit a high degree of crosslinking, providing outstanding characteristics in thermal stability, chemical resistance and electrical properties [1]. In addition, they show low shrinkage and high adhesive strength towards many materials. This makes them favorable for many applications, where the performance of thermoplastic foams is not sufficient [2]. They are mainly applied in marine, aeronautic and space applications as well as electronics [3,4].

In order to get a foamed or porous epoxy material, different routes are available. One possibility is the use of void templates. Here, preferably hollow spheres made of glass, ceramics, metals or polymeric materials are mixed into the liquid resin system [5,6]. The high viscosity of the composite mixture is a major drawback, leading to difficulties during processing with regard to homogenization and entrapped air [6]. To keep the viscosity low, reactive diluents are used, which influence the microstructure [7] and often notably reduce the glass transition temperature (T_g_) of the system [8]. On the other hand, this technique requires no special attention to the reaction kinetics of the matrix system compared to the routes involving blowing agents.

Another way is the use of physical or chemical blowing agents (BAs). In this case, the control of the reaction kinetics is a crucial factor. Their application is more challenging, as the viscosity development is highly dynamic from low molecular monomers to an infinite thermoset polymeric network. At the beginning, the diffusion rate is very high, while in the final stage, no proper cell development or expansion is possible. Thus, the systems require proper adjustment between curing kinetics and the BA activity.

Physical blowing agents (PBAs) are typically applied in a batch autoclave process or mechanical whipping [9]. These techniques can be applied well, as the foaming can be induced independent from the curing reaction. A quick pressure drop or temperature rise induces the expansion within seconds. Ito et al. investigated the influence of the gel fraction and molecular weight between crosslinks (Mc) on cell morphology in a temperature-quench physical foaming method with CO_2_. They determined a certain threshold Mc value which should exceed the size of the CO_2_ bubble nucleus. In addition, the complex modulus was found to allow for controlling the cell size [10]. Lyu et al. used a two-step batch process with CO_2_ to adjust the pre-curing degree of the system before foaming was induced. By adjusting the same pre-curing degree, independent from pre-curing conditions with regard to time and temperature, comparable foam properties were achieved [11].

Chemical blowing agents (CBAs) require a comparably long time to foam, depending on the decomposition kinetics, starting at a defined decomposition temperature. Thus, the foaming goes along with the curing epoxy system and both kinetics are important to consider. Takiguchi et al. investigated a DGEBA resin with an imidazole curing agent and azodicarbonamide as a chemical blowing agent (CBA). The systems were found not to foam without pre-curing, as the gas escaped before the system was able to build up a sufficient viscosity to entrap the gas. With increasing pre-curing time, especially beyond the gelation point, the systems revealed smaller cells and narrower cell size distributions [12]. Several other publications can be found which describe the beneficial effects of pre-curing on the foam morphology of epoxy foams [13,14,15,16].

**Figure 1:**
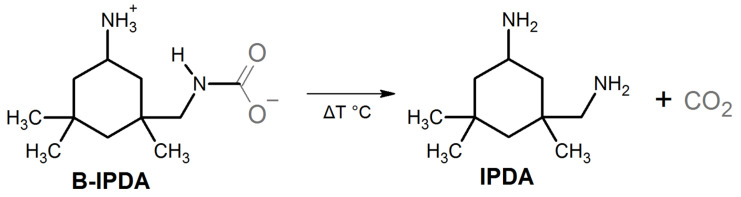
Decomposition reaction of isophorone diamine carbamate (B-IPDA) releasing the IPDA curing agent and CO_2_ blowing agent.

The main issue with regard to epoxy foaming are the blowing agents themselves. Most of the established blowing agents are harmful and/or raise safety concerns. The low boiling organic compounds used as PBAs [9] require high safety standards, while the CBAs often release harmful by-products [17]. Recent evaluations, for example, the “Registration, Evaluation, Authorization and Restriction of Chemicals” (REACH) or “Significantly New Alternatives Policy” (SNAP), lead to ongoing abandonment of many BAs. Among the BAs with the least concern, CO_2_ is one of the most well-established ones. It is widely applied for thermoplastic processing in gaseous and supercritical states. The research on its application in thermosetting systems is of high interest [10,11]. Among the CBAs, only a few systems are available which release CO_2_, such as carbonates and carbamates. Carbamates are suitable, as they release upon decomposition CO_2_ as a blowing agent and its basic amine, which can react with the epoxy resin to build up the thermosetting network. Figure 1 illustrates the decomposition reaction of isophorone diamine carbamate (B-IPDA).

In this example, the released IPDA acts as a curing agent while the CO_2_ acts as a blowing agent. Thus, for 100% carbamate systems, pre-curing is inapplicable because they decompose and cure simultaneously. The main advantage of this dual function is the fact that no undesired by-products are left in the foaming system by maintaining the original epoxy network composition as derived from the neat amine hardener and epoxy resin.

Several patents describe synthesis routes of carbamates and applications for epoxy foams made thereof, beginning in the early 1960s [18,19,20]. Recent applications were patented in the sector of expandable epoxy glue systems [21]. However, patents do not provide details on the specific effects taking place, and these details are required to understand and improve the foaming behavior.

Ren et al. recently provided a synthesis route for different CO_2_-loaded amines, provided as carbamates, and their basic properties, as well as the curing characteristics with DGEBA [22,23]. Furthermore, the foaming behavior was investigated by DSC, revealing different behaviors during foaming for the different carbamates, related to their basic amine structure. The foams were prepared in an adjusted one-step approach with 100% of carbamates, a nucleating agent and cell stabilizer. The resulting foam properties regarding the cell size and mechanical properties were dependent on the chosen carbamate. The smallest cell size of 111 ± 54 µm was achieved at a density of around 320 kg m^−3^ and irregular cell morphology [22,23].

Our previous studies with a focus on carbamate systems went into further detail with regard to influencing factors on the foamability without additives. One of the studies revealed that a higher viscosity of the basic epoxy resin is suitable for foam morphology development [24]. A further study focused on the synthesis and decomposition characteristics of three different carbamates. Basic dynamic foaming experiments at different temperatures revealed the importance of the balance between decomposition kinetics of the carbamate and the curing kinetics of the released amines, which change significantly with the foaming temperature [25]. In a further study, DGEBA and EN resin with 100% B-IPDA and flame retardants were studied along with 30/70_w_ IPDA/B-IPDA systems for pre-curing. It was found that the morphology can be improved by either pre-curing or additives. While the additives additionally increase the mechanical properties, pre-curing leads to a slight increase in the T_g_ [26]. A suitable application for these types of foam can be as core material in high-performance sandwich panels.

From the previous studies in the literature, beneficial effects of pre-curing on epoxy foaming with CBAs and PBAs are well described and some positive effects are stated for epoxy–carbamate foams but without further investigations.

This paper focuses on the effect of pre-curing on epoxy–carbamate foaming and suggests a way to enable improved morphology control by blending neat amine and its carbamate to enable pre-curing.

The aim of this study is to identify an optimized blend ratio and suitable pre-curing time thereof to achieve an improved foam morphology with small cell size. Therefore, detailed investigations on the rheological and curing behavior of selected foaming systems were carried out. The selected hardener blend of IPDA and B-IPDA was chosen, as it was found to provide suitable kinetics for both curing and foaming [25]. In addition, the B-IPDA-cured epoxy foams were found to provide suitable mechanical and temperature performance [26]. A DGEBA epoxy resin is cured with IPDA and B-IPDA at different blend ratios. The curing and rheological behavior is investigated and correlated to the resulting foam morphology with regard to cell size and shape. Furthermore, the effect of the pre-curing time on the morphology is investigated for a 30/70_w_ IPDA/B-IPDA blend.

## 2. Materials and Methods

### 2.1. Materials

Diglycidylether of bisphenol A (DGEBA, DER331, Olin, MI, USA) with a molecular weight (Mw) of 349.4 g mol^−1^, an epoxy equivalent weight (EWW) of 187 g mol^−1^ and a density of 1.16 g cm^−3^ was used as a resin system. IPDA (Aradur 22962, Huntsman, TX, USA) with a Mw of 170.3 g mol^−1^, an amino hydrogen equivalent (AHEW) of 42.6 g mol^−1^ and a density of 0.92 g mol^−1^ was used as received.

The CO_2_-loaded IPDA carbamate (B-IPDA) was synthesized by bubbling CO_2_ through an amine:EtOH solution and subsequent filtering and drying, as reported in our previous study. The reaction is illustrated in Figure 1 [25].

The B-IPDA had a Mw of 214.3 g mol^−1^, an AHEW of 53.6 g mol^−1^ and a density around 1.26 g mol^−1^. The decomposition temperature was around 70 °C and the CO_2_ content was determined to be 21 wt.% [25].

For foaming, an aluminum mold, made by the technical workshop at the University of Bayreuth, with dimensions of 30 × 30 × 10 mm³ and a resulting volume of 9 cm³, was used. As a release agent, Loctite Frekote 770-NC (Henkel, Duesseldorf, Germany) was used.

### 2.2. Methods

#### 2.2.1. Mixing

The dried carbamate was milled in a ball mill (Retsch PM 100, Haan, Germany) two times with five balls at 170 rpm^−1^ for 1 h. Afterwards, a stoichiometric amount of DGEBA and B-IPDA, corresponding to a weight ratio of 1:0.29 (DGEBA:B-IPDA), was pre-mixed by hand in the 50 °C pre-heated DGEBA. The dispersion was transferred to a 120 EH-450 three-roll mill (EXAKT Advanced Technologies GmbH, Norderstedt, Germany). The roll temperature was set 50 °C, roll speed to 200 min^−1^ and the gap sizes to 15 and 5 µm in gap A and B, respectively. Afterwards, the masterbatch (MB) was stored in pre-selected amounts required for 9 g of final foaming mixture (Table 1) in the freezer.

#### 2.2.2. Foaming

**Figure 2:**
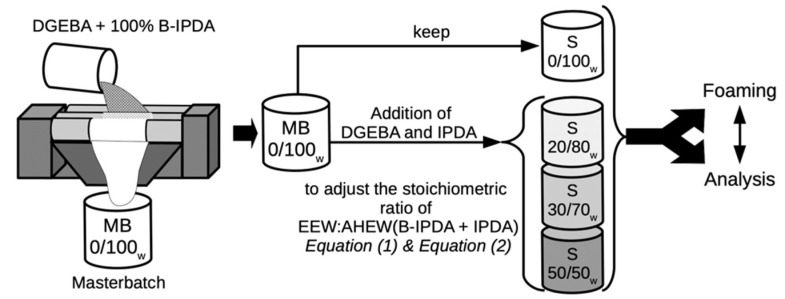
Workflow of sample preparation starting with masterbatch (MB) processing and further addition of DGEBA and IPDA in required amounts to meet new stoichiometric ratios with the desired blend systems of IPDA and B-IPDA, which were finally foamed and analyzed.

Before foaming, the mold was treated with the release agent and the MB was defrosted at 40 °C for 10 min. As the stoichiometric amount of B-IPDA (corresponding to DGEBA 0/100_w_ IPDA/B-IPDA) was already included in the MB, further amounts of DGEBA were added to the MB in order to achieve the required lower ratios of 20/80_w_, 30/70_w_ and 50/50_w_ IPDA/B-IPDA. The corresponding amount of IPDA was also added to the MB. The workflow is shown in Figure 2.

It is noted that due to the processing, the final IPDA:B-IPDA ratio is related to the stoichiometric AHEW by wt.%. This means that the 50/50_w_ blend contains the same weight of IPDA and B-IPDA to obtain the required AHEW, while the stoichiometric percentage is 44.1/55.9 (see Table 1). This is important for the subsequent calculation of the pre-curing degree.

For stoichiometric calculations, the AHEW_blend_ of the IPDA/B-IPDA hardener blend was calculated as shown in Equation (1):

(1) $${\mathrm{AHEW}}_{\mathrm{blend}}=\frac{1}{\left(\left(\frac{{\mathrm{ratio}}_{\mathrm{IPDA}}}{{\mathrm{AHEW}}_{\mathrm{IPDA}}}\right)+\left(\frac{{\mathrm{ratio}}_{B-\mathrm{IPDA}}}{{\mathrm{AHEW}}_{B-\mathrm{IPDA}}}\right)\right)}$$

The calculated AHEW_blend_ was further used to prepare the stoichiometric foaming systems as shown by Equation (2) for the resin amount:

(2) $$m_{\mathrm{Resin}}=m_{\mathrm{total}}\cdot \frac{{\mathrm{EEW}}_{\mathrm{DGEBA}}}{\left({\mathrm{EEW}}_{\mathrm{DGEBA}}+{\mathrm{AHEW}}_{\mathrm{blend}}\right)}$$

The required amount of hardener was calculated based on the resin (m_Resin_) and total amount desired (m_total_).

The dispersion of the further added DGEBA and IPDA proceeded for 1 min at 3500 rpm in a DAC 150 (Speed-Mixer, Hamm, Germany) mixing system. The required amount of each mixture was poured into the mold to 75 % of the cavity volume, allowing an expansion factor of 1.3. Therefore, the density of the mixtures ($\sigma_{\mathrm{mix}}$) was calculated based on the m_total_ and volume of the mixture (V_mix_) according to Equation (3):

(3) $$\rho_{\mathrm{mix}}=\frac{m_{\mathrm{total}}}{V_{\mathrm{mix}}}=\frac{\left(m_{\mathrm{IPDA}}+m_{B-\mathrm{IPDA}}+m_{\mathrm{DGEBA}}\right)}{(\frac{m_{\mathrm{IPDA}}}{\rho_{\mathrm{IPDA}}}+\frac{m_{B-\mathrm{IPDA}}}{\rho_{B-\mathrm{IPDA}}}+\frac{m_{\mathrm{DGEBA}}}{\rho_{\mathrm{DGEBA}}}}$$

The material required to fill the mold with the required amount for foaming (a_F_) is dependent on the volume which is desired to be filled (V_fill_), in this case 75% of 9 cm^3^, resulting in 6.75 cm^3^, finally calculated by Equation (4):

(4) $$a_{F}=V_{\mathrm{fill}}\;\cdot {\;\rho }_{\mathrm{mix}}$$

Due to the different compositions and densities of the mixtures, the a_F_, and thus expected density $(\sigma_{\exp}$), are different. The residual mixture was used directly for further characterization.

The theoretical CO_2_ weight content of the mixtures was calculated by the mass of contained CO_2_ which is related to the known CO_2_ content in the carbamate (wt.%_ca_), which is 21 wt.% for B-IPDA, by Equation (5):

(5) $$\mathrm{wt}.{\%}_{{\mathrm{CO}}_{2}}=\frac{m_{{\mathrm{CO}}_{2}}}{a_{F}}=\frac{{\mathrm{bI}}_{\mathrm{MB}}\cdot \mathrm{wt}.{\%}_{\mathrm{ca}}}{a_{F}}\;$$

The values are summarized in Table 1.

The filled mold was transferred to a PW 20 hot press (P/O/Weber, Remshalden, Germany) and set to 60 °C. This temperature was found to be suitable as it provides a certain gap between the decomposition temperature of the B-IPDA at 70 °C. After specified times of 1, 2, 3 and 5 h, the press was heated to the curing and foaming temperature of 180 °C which was found to be most suitable during pre-trials. To avoid an entrapping of the carbamate particles in the pre-cured network, a fast decomposition thereof is required. To increase the decomposition rate properly to the reaction rate of the IPDA, a temperature of 180 °C is required, as shown in our previous study [25].

The overall heating rate inside the mold was determined to be 7 °C min^−1^. After 1 h, the sample was transferred into a cooling press and left at room temperature. The specimen was finally demolded and characterized. The temperature was raised to 180 °C with same heating rate. One hour later, the mold was cooled down to room temperature and the foam was released.

#### 2.2.3. Characterization

i. Characteristics of IPDA/B-IPDA blend systems

The investigations on the curing behavior of selected systems were carried out by differential scanning calorimetry (DSC) with a DSC1 Star System (Mettler Toledo, Columbus, OH, USA) with 50 mL min^−1^ N_2_. The foaming process-related measurements were adapted to the process steps with dynamic heating ramps of 7 °C min^−1^ with corresponding isothermal steps at 60 °C and a subsequent isothermal step for 1 h at 180 °C.

The pre-curing degree (D_P_) was calculated from the released energy at the specific point in time (E_s_) and a reference value (E_ref_) corresponding to 100% curing by Equation (6):

(6) $$D_{P}=\frac{E_{s}}{\left(\frac{E_{\mathrm{ref}}}{100}\right)}$$

E_ref_ refers to a DbI 100/0 system gained from a dynamic measurement from −30 to 300 °C at 10 °C min^−1^, corresponding to −524 J g^−1^ [25], as the pre-curing is not affected by the B-IPDA decomposition.

The rheological investigations were conducted with a Physica MCR 301 (Anton Paar, Graz, Austria). A plate setup of 25 mm and 1 mm gap size with a deformation of 10% and a frequency of 1 rad s^-1^ was applied. During the measurements, the normal force (F_N_) was set to zero, allowing the tool to vary the gap size depending on expansion. The measurements related to the foaming process were adapted with dynamic heating ramps of 7 °C min^−1^ and corresponding isothermal steps at 60 °C with a subsequent isothermal step for 1 h at 180 °C.

ii. Properties of received epoxy foams

The density measurements were performed in distilled water with an AG245 analytical balance (Mettler Toledo, Columbus, OH, USA) with the use of a density kit for AG balances by the Archimedes principle according to DIN EN ISO 845.

The T_g_ of the systems was evaluated by dynamic mechanical analysis (DMA) with a sample size of 30 × 10 × 3 mm with an RDA III (TA Instruments, New Castle, DE, USA). The specimen was heated from 25 to 300 °C with a heating rate of 2 °C min^−1^ and measured with a frequency of 1 Hz.

To obtain the foam morphology, a cryogenic fracture was sputter-coated with gold in a SPUTTER Coater 108auto (Cressington Coating systems, Dortmund, Germany). The SEM pictures were recorded with a Leo 1530 (Zeiss, Oberkochen, Germany).

The cell size was evaluated with ImageJ version 1.51m9.

## 3. Results and Discussion

### 3.1. Influence of the Neat Amine Content

**Figure 3:**
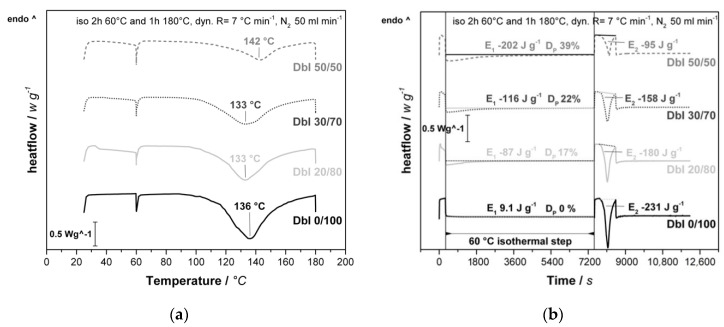
Results from the process-related DSC analysis of DbI systems with an IPDA/B-IPDA blend ratio of 0/100_w_, 20/80_w_, 30/70_w_ and 50/50_w_. (**a**) DSC thermograms plotted against temperature with indicated peak temperatures. (**b**) DSC thermograms with indicated peak energies (E_1_ and E_2_) and resulting pre-curing degree (D_P_) plotted against time with indicated 60 °C isothermal step for pre-curing.

To investigate the foaming behavior with different DbI blends, an experimental series with ratios of 0/100_w_, 20/80_w_, 30/70_w_ and 50/50_w_ IPDA/B-IPDA was conducted. The resulting CO_2_ content for each ratio was calculated according to Equation (5) and is presented in Table 1. The corresponding DSC thermograms reveal the curing behavior of the different blend systems and are presented in Figure 3.

In the DSC experiments, two exothermal peaks can be observed. The first one in the range of 20 to 60 °C (Figure 3a) is related to the pre-curing of the systems, while the second one is related to the decomposition of the carbamate and further curing with the released IPDA. The onset is dependent on the blend system and can be observed at around 90 °C, whereas the actual reaction is expected to start at a lower temperature, as found in our previous study [25]. The peaks are in the range between 133 and 142 °C, depending on the blend ratio. The shift can be explained by the diffusion of the released IPDA, which is highest at DbI 20/80_w_ and DbI 30/70_w_, while the high pre-curing degree at DbI 50/50_w_ lowers the diffusion.

As the isothermal step of the experiment does not allow a direct evaluation of the peaks, a plot versus time (Figure 3b) provides more accurate insights to the effects during pre-curing. It is observed that with increasing IPDA content, the exothermal energy in the pre-curing step, as well as the duration, increases. It is noted that the DbI 0/100_w_ sample exhibits a negligible endothermal energy, which contributes to the energy required to adjust the temperature in the initial state due to lower heat conductivity with the high filler content of B-IPDA. The pre-curing degree does not reach the theoretical maximum value calculated from the stoichiometric amount of IPDA for all systems, as shown in Table 1.

The second peak reveals a decreasing energy value of the main curing and foaming step by increasing the blend ratio. This can be explained by the increasing pre-curing degree which reduces the amount of remaining IPDA released by the carbamate. The increasing peak size can be attributed to neat IPDA remaining after 2 h, which reacts further before the B-IPDA decomposes. The determined energy values in the range up to 180 °C are summarized in Table 2. A small amount of energy released in the final isothermal step at 180 °C can be observed for all samples, but is not investigated further as the foaming is already completed at this point. The total energy released cannot be determined due to the mixture of dynamic and isothermal steps in the experiment. However, the trend confirms the expectation of a reduced total energy release with increasing B-IPDA content due to the endothermal decomposition [24,25,26].

**Figure 4:**
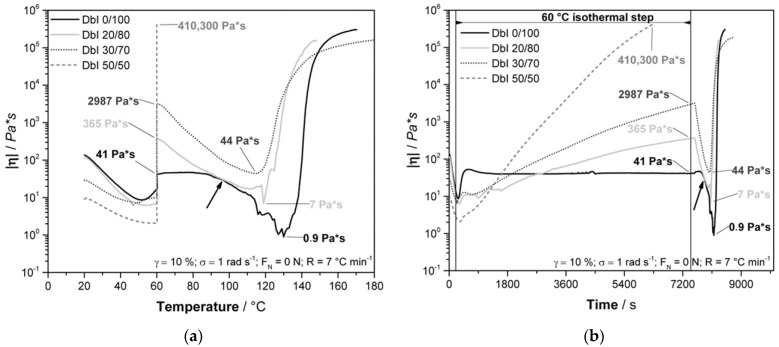
Results from the process-related rheological analysis of DbI systems with an IPDA/B-IPDA blend ratio of 0/100_w_, 20/80_w_, 30/70_w_ and 50/50_w_. (**a**) Rheological results dependent on the temperature, (**b**) rheological results dependent on the time with indicated 60 °C isothermal step, with both indicated viscosities after pre-curing and minimum viscosity during foaming.

The pre-curing effect and trends are also observable in the rheology experiment, as shown in Figure 4.

The overall trend of the rheological experiments matches well with the DSC results. The observed differences between DSC and rheology can be explained by the methodology of the measurement, as the DSC focuses on changes in the energy flow, while the rheometer physically applies shearing to the system and detects the response. In general, the initial viscosity decreases with increasing amounts of IPDA due to its lower viscosity and less solid B-IPDA filler content. The viscosity is increased up to different levels during isothermal pre-curing, with a subsequent drop caused by further temperature increases and carbamate decomposition, releasing CO_2_ which provides plasticizing effects (Figure 3a). Afterwards, the rising foam structure disturbs the measurement, resulting in different slopes and further noise due to some material being pushed out of the measurement gap. After an initial re-orientation of the system, the DbI 0/100_w_ sample shows a constant viscosity value of around 41 Pa × s during the 2h pre-curing time, which is lower compared to the initial viscosity at 25 °C with 136 Pa × s due to the temperature influence (Figure 4b). Thus, combined with the DSC results, the latent characteristics of the B-IPDA can be confirmed.

The minimum viscosity was found at temperatures around 115–130 °C, caused by the temperature increase and the CO_2_ release. As the released amine starts to cure the system, the viscosity increase is accelerated by temperature, once most of the B-IPDA decomposes and the CO_2_ either escapes or is entrapped. The effect of curing and CO_2_ release can be observed by the measurement noise at temperatures above 90 °C (Figure 4a) and the time beyond 7650 s (Figure 4b). For DbI 0/100_w_, the measurement noise is very strong up to 130 °C, revealing the carbamate nature of curing and blowing at the same time. Only when most of the B-IPDA is decomposed and no further CO_2_ disturbs the network formation can a continuous increase be detected. The observed slight increase at DbI 0/100_w_ before the drop in viscosity is related to an initial oligomer or network formation of the released IPDA, which overcomes the bubble nucleation at the initial stage. At around 98 °C, the viscosity of DbI 0/100_w_ matched the viscosity of DbI 20/80_w_ for a short time and further decreased. In fact, the pre-cured polymeric structure of DbI 20/80_w_ behaved pseudoplastically, so its viscosity was reduced with a higher temperature and also because of the presence of released IDPA and CO_2_. The more significant reduction in viscosity of DbI 0/100_w_ is an evidence that it is still in a mainly monomeric state compared to the pre-cured network of DbI 20/80_w_. This exhibits the advantage of blending neat amine and carbamate. The pre-cured network of the DbI 20/80_w_ leads to higher viscosities and modulus of the network, lowering the noise effect. This indicates a better ability to entrap the CO_2_ with less cell fracturing. At DbI 30/70, the noise effect cannot be seen. This leads to the conclusion that the 30/70_w_ blend ratio leads to the best matching viscosity and modulus to keep the CO_2_ in a stable pre-cured network for foaming. The increased pre-curing degree observed from DSC increases with the amount of neat IPDA in the blend, resulting in a lower viscosity drop because the polymeric network size increase and more entanglements and crosslinks are likely to stabilize it. This also leads to an earlier minimum of viscosity after pre-curing. In case of the DbI 50/50_w_ blend, even a full gelation of the network can be observed during the 2 h pre-curing time, leading to an abortion of the measurement. The fact that the system is still able to foam leads to the conclusion that the network reveals a viscous behavior during foaming at temperatures up to 180 °C.

**Figure 5:**
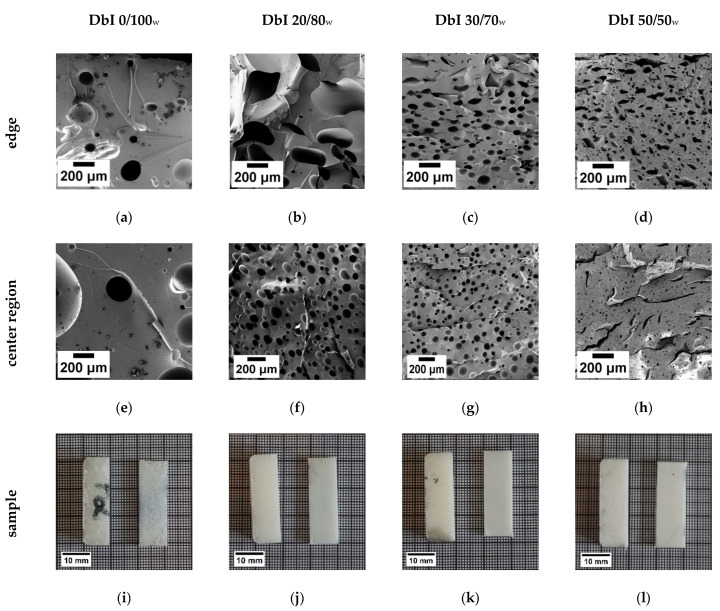
SEM pictures of the DbI systems with blend ratios of 0/100_w_ (**a**,**e**,**i**), 20/80_w_ (**b**,**f**,**j**), 30/70_w_ (**c**,**g**,**k**) and 50/50_w_ (**d**,**h**,**l**). The edge region is presented in the images (**a**–**d**), the core region in (**e**,**f**) and the foam sample in (**i**–**l**) from top side (**left**) and cross section (**right**).

The findings match well with the morphology of the foamed samples, as observed from the SEM images and photos in Figure 5.

In addition to a compact surface caused by the fast reaction of the neat IPDA on the hot surface of the mold, a reduction in cell size can be seen (Table 2). In systems containing neat IPDA, the surface of the foam cures immediately upon heating, resulting in a closed foam skin [4,24]. At the edges, the cells are irregularly deformed due to the fast-cured surface and the limited cell expansion in some directions, pushed by the expanding center region. The center region shows the desirable spherical morphology for DbI 0/100_w_, DbI 20/80_w_ and DbI 30/70_w_, as a uniform expansion is possible in all directions and the curing is controlled by the B-IPDA decomposition. The absence of pre-curing for the DbI 0/100_w_ system leads to a coarse foam structure. This results in a comparably large cell size and deviation, as well as remarkably large non-foamed areas and results in a low cell density. This is caused by the fact that the CO_2_ cannot be stabilized properly in the matrix with a viscosity minimum around 9 Pa × s (Figure 4) and the cells collapse and/or merge. With increasing neat IPDA content and consequently higher pre-curing degree and viscosity, the samples show a more homogeneous cell morphology. This can be observed from the lower deviations of the cell size and increases in cell distribution shown in Table 2. The systems can stabilize the nucleated cells better and prevent their coalescence. For DbI 50/50, an edge region with irregularly formed, bigger cells and a cracked morphology with small cells at the center region can be observed. The origin of the cracks in the structure contributes to the decomposing B-IPDA in the already highly viscous and initially crosslinked matrix, as revealed in Figure 4. The built-up pressure induced by CO_2_ release damages the pre-cured polymeric network, resulting in microcracks throughout the whole foam morphology. Due to the simultaneous release of IPDA, the system cures and crosslinks further, freezing the cracked structure.

For cell size evaluation, the center regions of the foams were analyzed. The cell size and foam density are also presented in Table 2.

The adjusted density was reached for all systems except the DbI 50/50_w_ which exhibits a slightly increased density. As the center region was taken for density measurements, the poor foam development thereof in the 50/50_w_ sample can be seen. It should be expected that the foaming does not influence the T_g_. However, the results observed from DMA are different. This effect is also reported in the literature for other epoxy foams [23]. While the DbI 0/100_w_ system exhibits the lowest T_g_, the value increases for the DbI 30/70_w_ blend, being closest to the non-foamed value, then drops for the 50/50_w_ blend again. As all the systems are fully cured and no further curing effects were observed, this can be explained by the overall thermoset network with increased defects resulting from incomplete network formation during the foaming process. With ratios of 20/80_w_ and 30/70_w_, the reaction kinetics and viscosity allow CO_2_ diffusion and bubble formation in the pre-cured polymeric structures, which disturb the further ongoing network formation. This effect is most striking for the 0/100_w_ system due to the simultaneous curing and blowing without a pre-formed network [23]. In the case of the 50/50_w_ ratio, the high pre-curing and fast gelation lowers the diffusion of the released IPDA. The additional bubble and crack formation can be seen to hinder the proper network formation or even weakens its structure. The overall influence of the porous structure on the storage modulus G′ at 30 °C can be seen when compared to the non-foamed DbI 100/0_w_ sample which exhibits a value nearly three times higher (Table 2). The G′ was found to reflect the trend of the density in general. comparing DbI 20/80_w,_ 30/70_w_ and 50/50_w_. However, the DbI 0/100_w_ value is higher compared to the DbI 50/50_w_ value and the density is lower. As an explanation, the big non-foamed areas compared to the more homogeneous, but cracked, structure in DbI 50/50_w_ can be seen. It can be concluded that the DbI 30/70_w_ system reveals the best performance with regard to rheological behavior and kinetics for a 2h pre-curing time. The DbI 30/70_w_ system was chosen for further investigations.

### 3.2. Influence of the Pre-Curing Time

**Figure 6:**
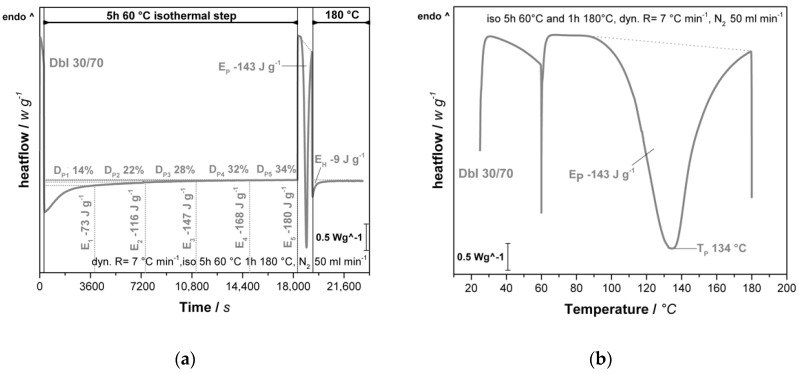
DSC investigations of the DbI 30/70_w_ system pre-cured for 5 h at 60 °C with process-related heating rates showing (**a**) time-dependent heat flow with indicated energy (E_x_) released at specific pre-curing times with corresponding calculated pre-curing degree (D_PX_) in a 5 h pre-curing experiment. E_p_ corresponds to the energy released at the peak of the dynamic step and E_H_ to the energy released at 180 °C (**b**) Temperature-related heat flow with indicated E_P_ and reaction peak temperature (T_P_).

To investigate the influence of the pre-curing time, the DbI 30/70_w_ system was used for further DSC and rheology measurements. It should be kept in mind that the stoichiometric amount of hardener in this system is shared between neat IPDA and B-IPDA. For a 30/70 wt.% ratio between IPDA and B-IPDA, a stoichiometric ratio of 35.2/64.8% is found. The corresponding diagrams of the process-related DSC are presented in Figure 6.

**Figure 7:**
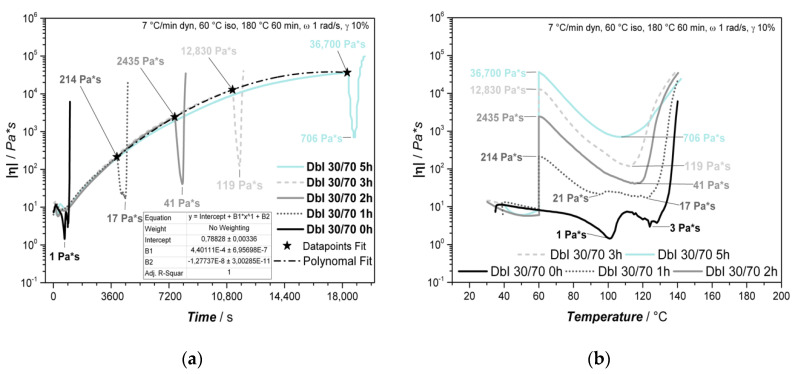
Investigations of the DbI 30/70_w_ system with different pre-curing times at 60 °C with process-related heating rates showing different (**a**) time dependence of viscosity for different foaming experiments with polynomial fit of maximum viscosities after pre-curing and (**b**) temperature dependence of viscosity for different foaming experiments, plotted up to 140 °C due to increased noise effects at higher temperatures.

In Figure 6a, the DSC evaluation shows the expected D_P_ values for different pre-curing times. After 5 h at 60 °C, the value converges close to the theoretical maximum D_P_ of 35.2%, considering an additional curing during the heating ramp up to 60 °C. The value calculated after 2 h matches well with the experiment shown in Figure 3b. The stability of B-IPDA at a given temperature leads to negligible release of IPDA in the timescale. The E_P_ is slightly lower compared to the 2 h experiment (Figure 3), as less neat IPDA remains after 5 h of pre-curing which contributes to this curing step. The T_P_ was found in the same range compared to the 2 h experiment. The results of the rheological experiments are shown in Figure 7.

**Figure 8:**
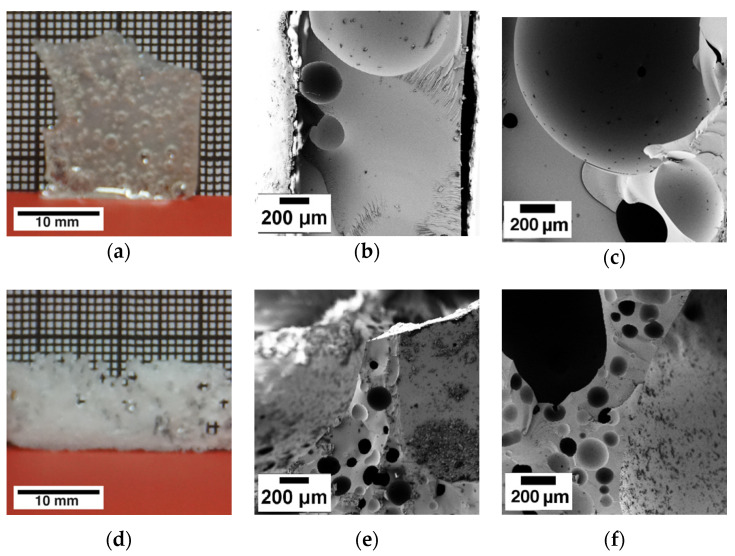

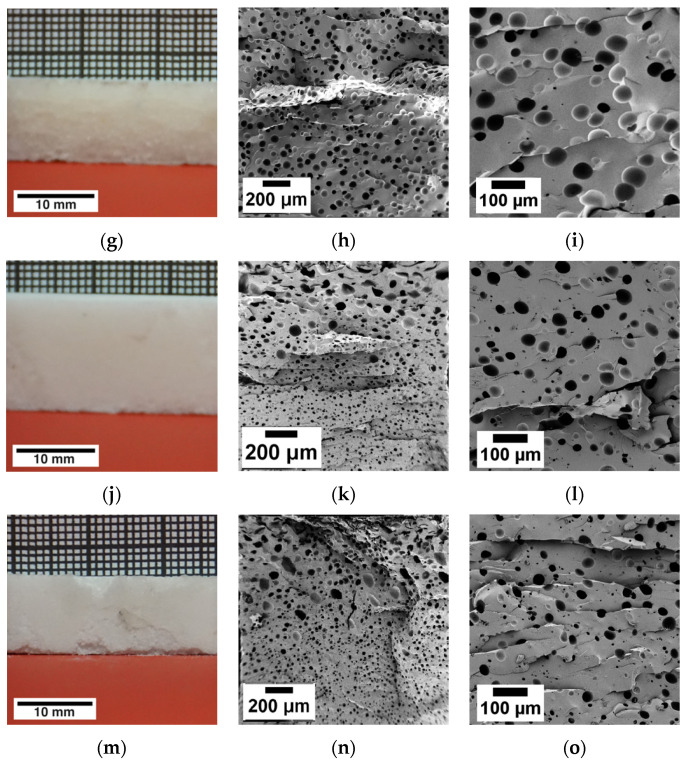
Pictures of the DbI 30/70_w_ systems which were pre-cured for different times of (**a**–**c**) 0 h; (**d**–**f**) 1 h; (**g**–**i**) 2 h; (**j–l**) 3 h and (**m–o**) 5 h at 60 °C and further cured and foamed for 1 h at 180 °C. The samples in the photos are orientated in expansion direction, except (**a**), which shows the top side view due to low expansion and to illustrate the bubbles formed, and (**b**), which shows the side view to illustrate the low expansion.

The slowdown of the reaction due to the IPDA consumption and network increase can be confirmed with Figure 6a. With increasing pre-curing time, the viscosity asymptotically approaches a constant value, as can be seen by the polynomial fit. This behavior is due to the limited content of neat IPDA in the hardener blend as well as the stability of B-IPDA at given conditions, limiting the amount of networking points. This behavior is typically observed for thermoset networks, as the molecular weight of the polymeric structures does not increase in a linear way due to crosslinking effects. Figure 6b reveals the temperature dependence of the viscosity from 60 to 140 °C. Experimental data are cut off at 140 °C due to the increasing effect of noise disturbing the measurement at higher temperatures. The effects taking place during the temperature increase are the same as discussed before. The temperature-induced chain mobility results in a softening of the pre-cured network and a decrease in viscosity. In addition, the decomposition of B-IPDA releases CO_2_ which acts as a plasticizer and further released IPDA causes further curing. Without pre-curing, a minimum viscosity of 1 Pa*s is detected at around 100 °C. Due to the IPDA content, the already formed network shapes the viscosity decrease effect differently compared to the experiment in Figure 4a. An initial drop resulting from the CO_2_ release is counterbalanced by an increase where further curing and CO_2_ escape are assumed. The further released CO_2_ lowers the viscosity again, before the system begins to cure fast at around 130 °C. At a pre-curing time of 1 h, the viscosity reaches a low-level region between 100 °C and 125 °C. This region becomes smaller with increasing pre-curing time and the minimum is shifted to lower temperatures. As a longer pre-curing time results in an enhanced polymeric structure with entanglements or even an initial network formation, a better stabilization of the nucleated cells due to a higher pre-curing degree can be assumed. The resulting samples and morphologies observed by SEM from the foaming experiments are shown in Figure 8.

With increasing pre-curing time, an improvement of the foaming behavior in general and a reduction in cell size can be observed. A direct kick to 180 °C leads to a poorly foamed sample with negligible expansion, as the kinetics of decomposition and curing do not match properly. Similar results were observed in our previous study [25]. The increasing pre-curing time contributes to the oligomer and polymeric structure formation, which increases the viscosity, leading to cell stabilization and prevention of cell collapse or coagulation. Thus, more cells can be stabilized and the cell size decreases while the cell distribution increases. This effect is similar to the melt strength of thermoplastic foam materials which is improved by longer chains and branching. The characteristics of the obtained foams are summarized in Table 3.

As shown in Table 3, the cell size of the foams decreases while the cell distribution increases with pre-curing time. The foam with 0 h pre-curing exhibits the highest density due to the high ratio of compact matrix with some entrapped bubbles, allowing no proper evaluation, as seen in Figure 8a. A bulk DGEBA-IPDA system exhibits a density of around 1105 kg m^−3^ [25]. The lowest density value is observed for the 1 h pre-curing sample. This is due to the low viscosity, while still being able to keep some CO_2_, as observed from the plateau in Figure 7b. However, due to the CO_2_ pressure in the closed mold, material was pushed out. Thus, the filling degree was lowered along with the resulting density. The foams which were pre-cured for 2–5 h were able to stabilize the CO_2_ bubbles and withstand cell coalescence or diffusion of the CO_2_ to the cavity, allowing them to withstand a push-out. Consequently, the target density of around 860 kg m^−^³ was reached. The T_g_ can be seen as constant, indicating no effect resulting from pre-curing. The storage modulus G′ at 30 °C was found to increase with the pre-curing time of 2–5 h. As an explanation, the higher pre-curing degree of the network can be seen, as well as the increasing amount of non-foamed areas between the cells. The trend is also confirmed by the highest brittleness of the 5 h pre-cured sample, as indicated by Figure 8m, where some parts were breaking out of the sample during preparation. This issue was less significant in the 2 h and 3 h pre-cured samples. In summary, the DbI system is most suitable in a two-step process with pre-curing between 2 h and 3 h at 60 °C to achieve homogeneous cell sizes below 100 µm.

## 4. Conclusions

Within this study, the foam morphology of DGEBA–carbamate epoxy foams was successfully improved by pre-curing of DGEBA epoxy resin mixed with different IPDA/B-IPDA blend ratios. In addition, an optimized time window was evaluated for the 30/70_w_ system. The decomposition of B-IPDA at temperatures above 70 °C causes an instant release of CO_2_ and the amine curing agent simultaneously and thus enhances the process control. It was shown that at a fixed pre-curing time of 2h at 60 °C, the cell size decreases when increasing the amount of neat IPDA in the blend ratio. Furthermore, no decomposition of the B-IPDA in a 2 h time range at 60 °C was confirmed for the DbI 0/100_w_ system. DbI 50/50_w_ exhibits partially open-celled and damaged structures. This could be explained by an excessive degree of pre-curing and a very high viscosity at the foaming step. After 2 h of pre-curing time at 60 °C, the DbI 30/70_w_ mixture revealed the best results with regard to processing as well as cell size, and led to a glass transition temperature up to 151 °C. The storage modulus G′ was found to reflect the general trend and decrease with the density. An exception was found when comparing DbI 0/100_w_ and DbI 50/50_w_, where the inferior morphology of DbI 0/100_w_ led to more non-foamed areas at a lower density which increased the modulus while the cracked structure of DbI 50/50_w_ lowers the modulus. Thus, DbI 30/70_w_ was chosen to investigate the factor of pre-curing time. The cell morphology improves from a solid epoxy sheet with some entrapped bubbles at 0 h pre-curing time to an inhomogeneous foam at 1 h pre-curing time. A further increase to 2 h and up to 5 h reveals a decrease in cell size and increased homogeneity in cell size. However, a notable embrittlement can be observed for the 5 h sample. The developed polymeric pre-structures stabilize the nucleated cells and their growth. The latent properties of B-IPDA allow the limitation of the pre-curing degree to the chosen neat amine content, which was nearly achieved after 5 h. The storage modulus G′ slightly increases with the pre-curing time due to the better pre-curing network formation. In conclusion, pre-curing reveals a promising method for tailoring the foam morphology of epoxy–carbamate foams and enhancing the overall foam performance.

## Figures and Tables

**Table 1:** Required amounts of materials for the different foaming mixtures referring to 9 g total foaming mixture. As B-IPDA is already included in the masterbatch (MB), only DGEBA and IPDA are added for dilution and stoichiometric curing. For completion, the amount of B-IPDA in the specific amount of MB (bI_MB_) and its resulting CO_2_ content, as well as the related stoichiometric percentage of IPDA, are also indicated. In addition, the expected density $\left(\sigma_{exp}\right)$ resulting from a filling amount of 75 % (a_F_) is provided.

Sample	Name	MB	bI_MB_	CO_2_	DGEBA	IPDA		a_F_	$\sigma_{exp}$
		[g]	[g]	[wt.%]	[g]	[g]	[%_st_]	[g]	[kg m^−3^]
DGEBA 0/100_w_ IPDA/B-IPDA	DbI 0/100_w_	7.93	2.05	4.78	0	0	0	7.93	880
DGEBA 20/80_w_ IPDA/B-IPDA	DbI 20/80_w_	7.20	1.58	3.69	1.41	0.39	24.0	7.81	868
DGEBA 30/70_w_ IPDA/B-IPDA	DbI 30/70_w_	6.30	1.35	3.15	2.12	0.58	35.2	7.76	862
DGEBA 50/50_w_ IPDA/B-IPDA	DbI 50/50_w_	4.50	0.93	2.17	3.57	0.93	55.9	7.72	858
DGEBA 100/0_w_ IPDA/B-IPDA	DbI 100/0_w_	0	0	0	7.29	1.71	100	-	1105

**Table 2:** (Pre-)curing peak energies (E_1_ and E_2_) and resulting pre-curing degree (D_P_), as well as foam properties with regard to cell size (c_s_), cell distribution (c_d_), foam density $\left(\sigma_{f}\right)$ (process adjusted density is 860 kg m^−3^) and T_g_ (loss modulus G″ max. in DMA) as well as the storage modulus (G′) received at 30 °C from DMA (G′_30_) of the different blend systems.

System [wt.%]	E_1_ [J g^−1^]	D_P_ [%]	E_2_ [J g^−1^]	c_s_ [µm]	c_d_ [counts/10^6^ cm^−^^3^]	$\sigma_{f}$ [kg m^−3^]	T_g_ [°C]	G′_30_ [MPa]
DbI 0/100_w_	9.1	n.a.	−231	166 ± 190	n.a.	884	126	362.9
DbI 20/80_w_	−87	17	−180	61 ± 24	0.88	853	145	194.4
DbI 30/70_w_	−115	22	−158	48 ± 19	2.18	857	151	226.2
DbI 50/50_w_	−201	39	−95	4 ± 3	273	908	140	301.9
DbI 100/0_w_	-	n.a.	−524	-	n.a.	1105	158	1075.4

**Table 3:** DbI 30/70w foam properties with regard to pre-curing degree (D_P_), cell size (c_s_), cell distribution (c_d_), foam density ($\sigma_{f}$) (process-adjusted density is 860 kg m^−3^) and T_g_ (loss modulus G″ max. in DMA) as well as the storage modulus (G′) received at 30 °C from DMA (G′_30_) of the different blend systems.

Pre-Curing Time [h]	D_P_ [%]	c_s_ [µm]	c_d_ [counts/10^6^ cm^−3^]	$\sigma_{f}$ [kg m^−3^]	T_g_ [°C]	G′_30_ [MPa]
0	0	n.a.	n.a.	910	n.a.	n.a.
1	14	n.a.	n.a.	394	n.a.	n.a.
2	22	48 ± 19	2.9	857	151	206.4
3	28	21 ± 11	14.9	864	152	226.2
5	34	13 ± 8	36.9	852	150	281.1
